# Effective inhibition of dengue virus replication using 3′UTR-targeted Vivo-Morpholinos

**DOI:** 10.3389/fimmu.2024.1491230

**Published:** 2024-11-29

**Authors:** Mengwei Niu, Wenyanbo Yi, Zhuofan Dong, Xiaofeng Li, Xue Dong, Lifang Yu, Yao Han, Oujia Zhang, Ziyang Sheng, Jing An, Hao Li, Yansong Sun

**Affiliations:** ^1^ State Key Laboratory of Pathogen and Biosecurity, Beijing Academy of Military Medical Sciences, Beijing, China; ^2^ Department of Microbiology, School of Basic Medical Sciences, Capital Medical University, Beijing, China

**Keywords:** antisense oligonucleotides, antiviral, dengue virus (DENV), antisense therapy, nucleic acid therapy

## Abstract

**Introduction:**

Due to the impact of antibody-dependent enhancement and viral variation, effective vaccines or antiviral therapies remain lacking for the dengue virus (DENV). Nucleic acid drugs, particularly Vivo-Morpholinos (MOs), have emerged as a promising avenue for antiviral treatment due to their programmability and precise targeting, as well as their safety and stability.

**Method:**

In this study, we designed and developed 10 morpho-modified (octa-guanidine dendrimer) vivo-MO molecules that target each coding gene of DENV. Subsequently, we assessed the inhibitory impact of vivo-MOs on dengue viral RNA load utilizing qRT-PCR. Furthermore, we examined the inhibitory effect on the live virus through a plaque assay and the TCID50 assay.

**Results:**

We found that the vivo-3′UTR molecule targeting the 3′ untranslated region of the dengue virus exhibited the highest inhibitory rate against viral load. The vivo-3′UTR demonstrated 99% inhibition of dengue virus RNA and the inhibition of up to 98% of the live virus. Additionally, the targeted sequence was conserved among all four DENV serotypes, and treatment with 10 μM of vivo-3′UTR resulted in a reduction of viral titers for all four DENV serotypes by over 99.99%. Additionally, we revealed that pre-treatment with vivo-3′UTR had a notable preventive effect against viral infection.

**Conclusion:**

This study screened an effective vivo-MO target drug for the treatment of dengue virus infection, demonstrating low toxicity in mammalian cell lines, and proposed a novel preventive antiviral approach.

## Introduction

The dengue virus (DENV), a member of the *Flavivirus* family and a single-stranded RNA virus (1), is a significant source of mosquito-borne viral infections that impact human health (2, 3). DENV consists of four distinct antigen-associated virus serotypes (DENV-1–4) and can lead to three distinct pathological conditions: dengue fever (DF), dengue hemorrhagic fever (DHF), and dengue shock syndrome (DSS) (4). Currently, the worldwide prevalence of dengue fever is a significant concern, with approximately 390 million individuals being infected with DENV annually across 128 countries and regions. Of these cases, over 100 million individuals exhibit symptoms of DHF each year, with a mortality rate of 2.5% (5). Several studies have utilized chimeric structures to develop a quadrivalent attenuated live vaccine known as Dengvaxia^®^ (6), which elicits the production of protective neutralizing antibodies against four serotypes (7). However, additional research has demonstrated that the existence of cross-reactive antibodies targeting various DENV serotypes may exacerbate disease progression (8, 9), potentially leading to a more severe and fatal second infection with the dengue virus (5, 9, 10). Based on this impact, there is no effective means for the prevention and treatment of dengue virus infection at present (11, 12). Furthermore, with the ongoing mutation of the virus, conventional antiviral medications and vaccines may result in diminishing efficacy against the evolving virus strains. Consequently, there is a critical need to explore the development of programmable nucleic acid drugs with antiviral properties to combat dengue virus infections.

Antisense oligonucleotide (ASO) drugs exhibit advanced synthesis technology and robust programmability, rendering them a highly promising avenue for antiviral therapy (13, 14). ASO consists of chemically synthesized oligonucleotides, typically ranging from 12 to 30 nucleotides (15, 16), and it is a molecular drug that inhibits gene expression by sequence-specific binding to the target gene DNA or mRNA and regulates it at the gene level (17, 18). Currently, over 10 ASO drugs, such as Tofersen and Nusinersen, have received market approval, with numerous others undergoing clinical trials (19). Concurrently, some researchers have conducted chemical structure modifications on the ASO sequences or added specific sequences at both ends to improve its affinity for binding to the target sequence, making it resistant to the degradation of nucleases in the cell (20–22). In recent years, scholars have directed their attention toward morpholino-based therapeutics for DENV infections (23, 24). Holden et al. developed three phosphorodiamidate morpholino oligonucleotide (PMO) molecules specifically targeting DENV and conducted an analysis of their mechanism of action (25). Phumesin et al. pioneered the creation of the first *in vivo* morpholino oligomer (vivo-MO) targeting the 3′ terminus portion of the DENV gene region, showcasing its ability to bind to the DENV RNA target sequence and effectively inhibit virus production in A549 cell lines (26) and monocyte-derived dendritic cells (27). These findings indicate that targeting the 3′ untranslated region (UTR) can effectively suppress dengue virus replication. In contrast, our prior investigation utilized CRISPR systems directed toward 10 gene sequences of the dengue virus, revealing that targeting the NS3 gene with CRISPR systems resulted in significant inhibition of viral replication (28). This implies that the NS3 region of the dengue virus may represent a promising target for antiviral therapy. Vivo-MO has been documented to effectively inhibit the replication of DENV by directly binding to target mRNA within cells without the need for delivery vectors. Nevertheless, no researchers have screened the vivo-MO target sequence in the whole genome of the dengue virus; therefore, further research is needed to ascertain whether there exist more efficacious vivo-MO targets within the entirety of the dengue genome.

In this study, we conducted an analysis of the conserved sequences within each coding region of DENV and developed 10 *in vivo*-MO sequences targeting various conserved regions. Through the evaluation of 10 targets, we identified the most effective *in vivo*-MO target region (target 3′UTR) for inhibiting the dengue virus. Furthermore, we conducted a comparative analysis of the target sequence of vivo-3′UTR against the sequences of the four dengue virus serotypes and observed that this sequence is conserved across all four serotypes. Concurrently, the inhibition efficacy of vivo-3′UTR against these four dengue virus serotypes exceeded 99.99%. In addition, we assessed the antiviral efficacy of *in vivo*-MO under both pre-treatment and post-treatment conditions and found that early addition of vivo-Mo was effective in preventing dengue virus infection while still providing good suppression of dengue virus after infection. Thus, this study provided a new treatment strategy for dengue virus infection.

## Materials and methods

### Cell and viral culture

HepG2 cells (human liver cancer cell line) and Vero cells (African green monkey kidney cell line) were grown in Dulbecco’s modified Eagle’s medium (DMEM; HyClone™, Thermo Scientific, Waltham, MA, USA) with 10% heat-inactivated fetal bovine serum (FBS; Gibco™, Invitrogen, Carlsbad, CA, USA), 1% penicillin G (100 U/mL), and streptomycin sulfate (100 g/mL) at 37°C and 5% CO_2_. The A549 cells (human lung carcinoma epithelial cell line) were grown in minimum Eagle’s medium (MEM) (HyClone™, Thermo Scientific, USA) with 5% FBS, 1% penicillin G, and streptomycin sulfate at 37°C and 5% CO_2_. All cell lines present in this study were preserved in the State Key Laboratory of Pathogen and Biosecurity (Beijing, China). Three dengue virus serotypes—DENV-2 (DENV-2/16681 strain), DENV-3 (DENV-3-80-2 strain), and DENV-4 (DENV4-B5 strain)—were collected and stored at the Institute of the Military Academy of Sciences. The dengue virus serotype-1 (DENV-1 Hawaiian strain) was kindly provided by Dr. An Jing, Capital Medical University. All dengue viruses were propagated in Vero cells in MEM as previously reported. The harvested viral culture supernatant was filtered through a 0.2-μm filter, titrated by a plaque assay on Vero cells, then aliquoted, and stored at −80°C until further use.

### Design and synthesis of antisense oligonucleotide

A total of 1,201 complete or nearly complete DENV-2 genomes (genome length ≥10 kb) were downloaded from the National Center for Biotechnology Information (NCBI) database. All the DENV genome sequences aligned with MAFFT using the “auto” option. The alignment was imported into the Jalview software. A consensus nucleotide (which was shared by > 50% of non-gap characters at the given position) was assigned to each position in the alignment. In addition, to determine sites that can be efficiently targeted by vivo-MOs and that are conserved among four different DENV serotypes, the genomic RNA sequences were compared from four viral strains each representing one dengue serotype (GenBank: DENV-1/NC_001477.1, DENV-2/NC_001474.2, DENV-3/NC_001475.2, and DENV-4/NC_002640.1). Based on the conservative analysis result of dengue virus sequences, 10 vivo-MO sequences with a length of 28 bp were designed. The 10 most conserved regions were located ahead of nine individual protein-coding regions (i.e., Capsid, PrM, NS1, NS2a, NS2b, NS3, NS4a, NS4b, and NS5) and one UTR at the 3′ end (3′UTR). All antisense oligonucleotides were designed according to the genetic sequence of the DENV-2 (NCBI GenBank, NC_001474.2), and all antisense oligonucleotides were provided by 4A Biotech Co., Ltd. (Beijing, China).

The sequence of the vivo-MO is 5′-TCCCAGCGTCAATATGCTGTTTTTTGTT-3′ (vivo-3′UTR group), with the 10,609 to 10,636 nucleotides base of the DENV-2 genome being complementary. Non-targeted contrast (NT group) oligonucleotide sequence is 5′-CCTCTTACCTCAGTTACAATTTATA-3′; this sequence is a mutation intron in Mediterranean anemia, which is not shown in other organisms and is complementary to the sequence of Mediterranean anemia patients, and there is little complementary sequence in all other organisms, so the sequence does not cause any phenotypic changes. A Vivo-Morpholinos was comprised of a morpholino oligo with a unique covalently linked delivery moiety, which was comprised of an octa-guanidine dendrimer. It uses the active component of arginine-rich delivery peptides (the guanidinium group) with improved stability and reduced cost. All vivo-MOs were resuspended in nuclease-free water to obtain a 10-mM final concentration stock, which was stored at −80°C until required. All antisense oligonucleotide sequences are shown in **Supplementary Table S1**.

### Antiviral effect evaluation method

The Vero cells, HepG2 cells, and A549 cells were from the Institute of Microbiology of the Military Academy of Sciences, which were developed under the conditions of 37°C and 5% CO_2_. A 0.5-mL cell suspension containing (10^5^) cells per well was prepared and added to a 24-well cell culture plate. The cells were allowed to grow for 16 h until they formed a monolayer. For the experimental group, 200 μL of serum-free DMEM containing DENV-2 virus was added at a multiplicity of infection (MOI) of 0.1 (PFU number/cell) to each well. After 2 h of virus infection, the solution was removed and washed with phosphate-buffered saline (PBS). Then, a 10-μM concentration of vivo-MO diluted by Opti-MEM solution was added. Four hours later, 1 mL of DMEM supplemented with 2% serum, 1% penicillin–streptomycin, and 1% glutamine was introduced to each well. The supernatant was collected at 24 h, 48 h, and 72 h post-treatment.

### RT-qPCR experiment

After 24 h, 48 h, and 72 h of virus infection, the cell supernatant was obtained, nucleic acid was extracted, and the gene content of the dengue virus was detected by RT-qPCR. The forward primer of RT-qPCR was DENV-F (5′-CTTACAAATCGCAGCAACAA-3′), and the reverse primer was DENV-R (5′-GTCTTTCCCAGCGTCAATAT-3′). RT-qPCR was performed using the One-Step PrimeScript™ III RT-qPCR Mix Kit (RR600A, TAKARA, Maebashi, Japan), the results of the Ct value were calculated, and the statistical analysis was carried out. Each reaction mixture contained 0.5 μM primers, 12.5 μL One-Step PrimeScript III RT-qPCR mix of RNA, 0.5 μM probe, 2 μL of RNA, and ddH_2_O up to 25 μL. The thermal cycling procedure was 52°C for 5 min and 95°C for 10 s, 40 cycles at 95°C for 5 s, and 60°C for 30 s. All RT-qPCR experiments included quality controls comprising DNase/RNase-free water instead of RNA template [non-template control (NC)] in each run. To quantify the DENV RNA, the DENV-2 RNA reference material (S3, Guangzhou BDS Biological Technology Co., Ltd.) was used as a standard to quantify DENV RNA that was extracted from the supernatant of the DENV-infected Vero cells, thus obtaining the RNA copies in 1 mL; this quantified DENV RNA was used as the standard throughout our experiments.

### Cell viability experiment

Vero cells were seeded into a 96-well plate at a density of 10^4^ cells per well, with each well containing 100 μL of 10% DMEM culture medium, and incubated at 37°C. Once the wells reached approximately 70% confluence, the DMEM culture medium was removed, and the cells were washed with PBS. Each well received 100 μL of vivo-MO at the same concentration as the target, with different sets diluted using Opti-MEM, while the control group received only the Opti-MEM solution. After 4 h, 200 μL of 2% DMEM was added to each well to promote slow cell growth. After 24 h, 48 h, and 72 h of addition of vivo-MO, the culture medium was switched to serum-free and phenol red-free medium to avoid potential interference from serum hormones or phenol red, and then the Cell Counting Kit-8 (CCK-8) solution was added at a volume ratio of 10:1 and incubated for 1 h. Subsequently, the absorbance [unit is optical density (OD)] at 450 nm of each sample was determined using a microplate reader (SpectraMax i3X, Molecular Devices, San Jose, CA, USA) to assess cell viability.

### Plaque experiment

A 1% solution of methyl cellulose (100:3 of the NaHCO_3_ solution, 100:2 of the proportion of the amount of glutamine solution, 100:1 of the proportion of the HEPES solution, and 100:5 of the proportion of the serum) was prepared in advance and stored at 4°C. The Vero cells were seeded onto the 24-well plate with the appropriate concentration (10^5^ cells per well) to ensure that the cells could reach 90% fusion in 24 h. MEM with 2% FBS and 1% penicillin–streptomycin was used to dilute the virus fluid (10-fold serially dilute). Each well was added to 0.2 mL of the virus fluid of the different dilution gradients. The MEM with 2% FBS was made as a negative control well, and the known virus liquid was tested for positive control. The cell plate was placed at 37°C for 1 h to allow for virus attachment. During the period, the virus fluid was gently shaken every 15 min. After incubation for 1 h, the liquid in the well was completely removed. MEM containing 2% FBS was used for washing, and then the medium of the MEM that contained 1.2% methyl cellulose was covered. Incubation was achieved for 7–8 days in a 37°C and 5% CO_2_ incubator. After the culture medium was completely discarded, crystal purple dye was used for staining for 30 min at room temperature. The medium was washed and dried, the number of virus plagues was counted, and the drop degree of the virus was calculated according to the dilution.

### TCID50 assay

Following a 10-fold serially dilution, various experimental samples were added to Vero cells in 96-well plates and incubated at 37°C and 5% CO_2_ for 90 min to facilitate viral adhesion. Subsequently, the supernatant containing the viral medium was removed, and DMEM supplemented with 1% bovine serum albumin (BSA) and 2 mg/mL TPCK trypsin was added to each well. The cells were then incubated at 37°C and 5% CO_2_ for a duration of 3 days, during which cytopathic effects were monitored daily. Upon cessation of pathological changes, the viral titer was determined and expressed as logTCID50.

### Statistical analysis

All analyses were performed using the GraphPad Prism program version 9.1.0 (GraphPad Software Inc., San Diego, CA, USA). Data were compared by Student’s t-test or one-way ANOVA. p-Value <0.05 (*), p-value <0.01 (**), p-value <0.001 (***), and p-value <0.0001 (****) were considered statistically significant; ns means no significant difference. Results were expressed as means ± SEM from at least three independent replicates.

## Result

### Design and screening of efficient vivo-MO targeting the conserved regions of DENV

**Figure 1:**
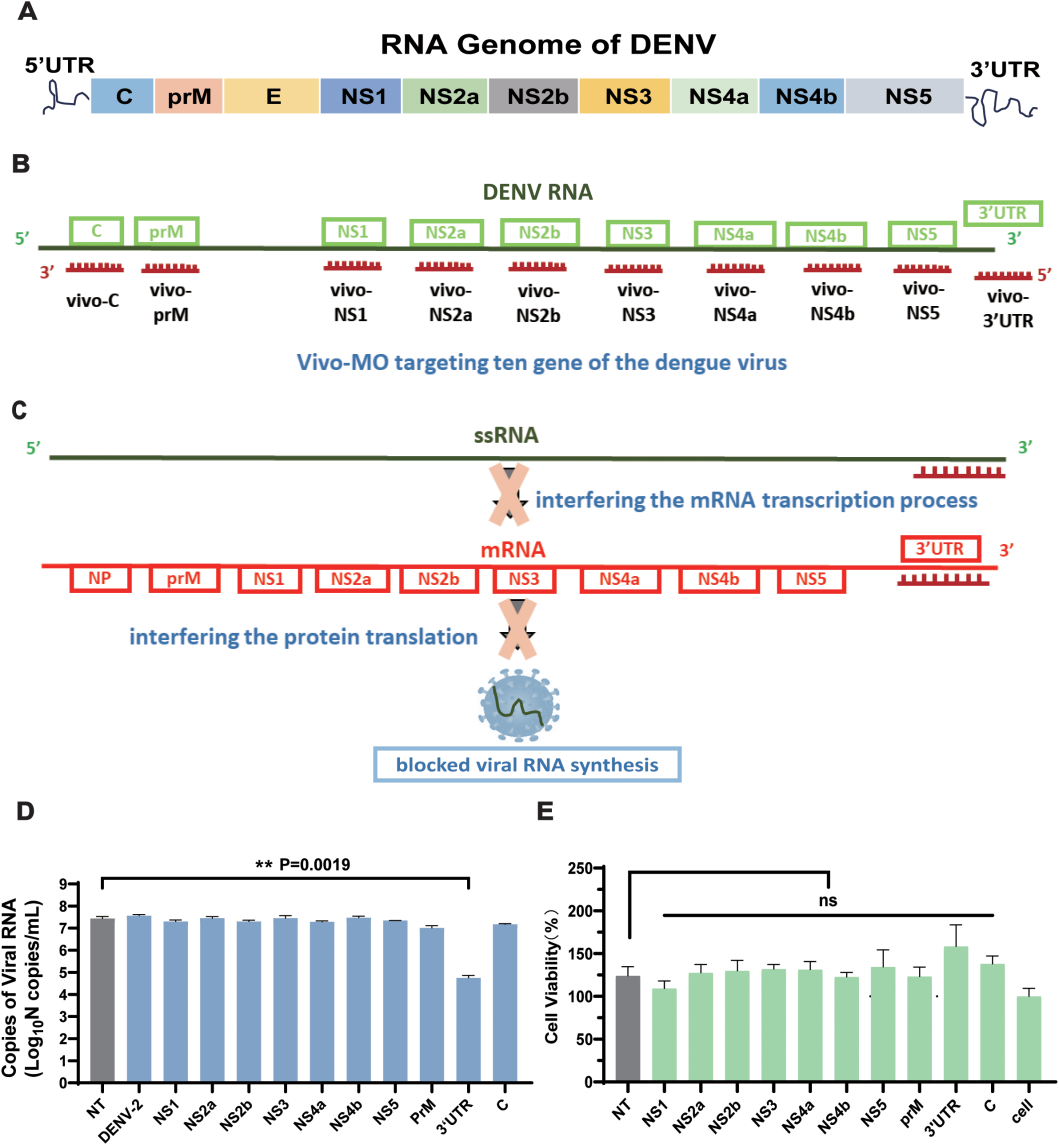
Structure schematic of DENV genome and the design schematic of the 10 vivo-MOs. **(A)** Schematic of RNA genome structure of DENV. **(B)** Design schematic of the vivo-MO targeting the genome of DENV. **(C)** Schematic diagram of vivo-3′UTR inhibiting dengue virus replication. **(D)** Screening results of 10 vivo-MOs were obtained in DENV-2 infection at 72 h. **(E)** Toxicity test of 10 vivo-MOs on Vero cell. Cell viability was measured by Cell Counting Kit-8 (Beyotime). n = 3 for each group, one-way ANOVA; **p < 0.01; ns, no significant difference; bars represent mean ± SD. DENV, dengue virus; MOs, Vivo-Morpholinos.

Based on the comparative analysis of dengue virus sequences (**Figure 1A**), 10 vivo-MO sequences with a length of 28 bp were designed, which target the 10 conserved regions of the dengue virus (**Figures 1B, C**). All vivo-MOs were comprised of a morpholino oligo with a unique covalently linked delivery moiety, which was comprised of an octa-guanidine dendrimer. It used the active component of arginine-rich delivery peptides (the guanidinium group) with improved stability and reduced cost (**Supplementary Figure S1**). Meanwhile, the non-targeted control (NT) group was designed as the negative control group. Then, the inhibitory effect of 10 targets for the dengue virus was evaluated compared with that of the other experimental groups; vivo-MO targeting the 3′UTR region (vivo-3′UTR) had the most significant inhibitory effect on the dengue virus, and the viral RNA load was reduced by 99.79% compared with that of the NT group at 72 h after viral infection (**Figure 1D**). Statistical analysis showed that all other targets except 3′UTR had no significant viral inhibition effect compared with the NT group. Next, the CCK-8 reagent was used to detect the effects of the 10 target drugs on cell viability, and the results showed that the viability of the cells in the vivo-3′UTR group was slightly higher than that of the control group, but the viability of all experimental groups was not statistically different compared with that of the NT group (**Figure 1E**). It was shown that the addition of vivo-3′UTR drugs had no significant effect on cell viability, and it was proved that the significant reduction of the number of dengue viral RNA loads caused by the vivo-3′UTR group was not caused by cell death.

### Screening of optimal concentration of the vivo-3′UTR to suppress DENV

**Figure 2:**
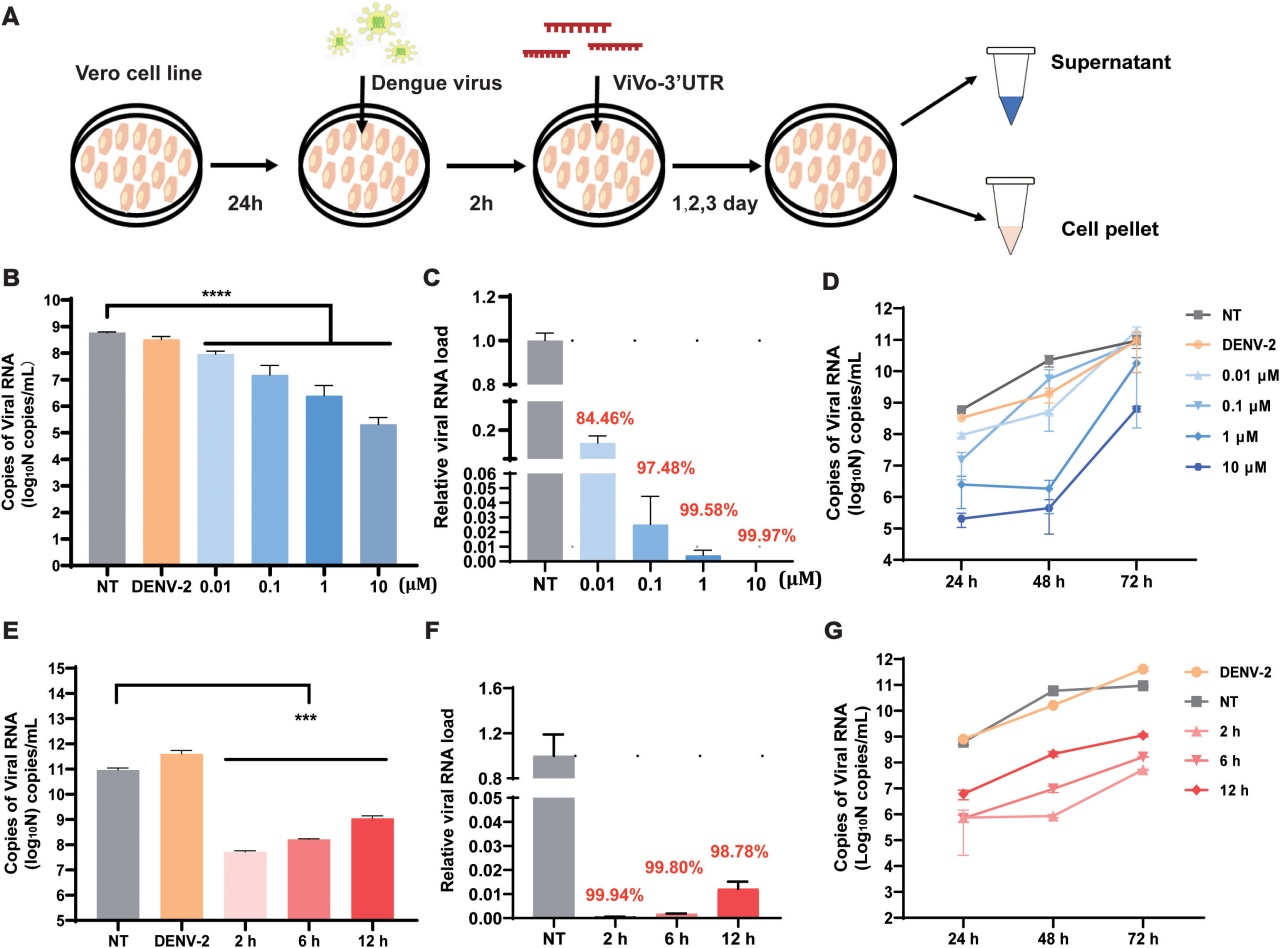
Determination of infection rate and optimization of vivo-3′UTR concentrations in DENV-2-infected Vero cell. **(A)** The vivo-3′UTR *in vitro* anti-dengue virus experimental process schematic diagram. **(B)** The inhibition result of different vivo-3′UTR concentration gradients (0.01 μM, 0.1 μM, 1 μM, and 10 μM) in DENV-2 infection at 24 h. **(C)** The relative viral RNA load of different vivo-3′UTR concentration gradients in DENV-2 infection at 24 h. **(D)** The inhibition result of different vivo-3′UTR concentrations in DENV-2 infection at 24 h, 48 h, and 72 h. **(E)** The result of different vivo-3′UTR administration times (2 h, 6 h, and 12 h) on DENV inhibition in DENV-2 infection at 72 h. **(F)** The relative viral RNA load of different vivo-3′UTR administration times in DENV-2 infection at 24 h. **(G)** The inhibition result of different vivo-3′UTR administration times in DENV-2 infection at 24 h, 48 h, and 72 h. n = 3 for each group, two-tailed Student’s t-test; ***p < 0.001; ****p < 0.0001; bars represent mean ± SD. DENV, dengue virus.

To verify the optimal suppression efficiency of the vivo-3′UTR, we set a four-concentration gradient (0.01 μM, 0.1 μM, 1 μM, and 10 μM) for antiviral experiments, and we used RT-qPCR to detect viral RNA load in the supernatant after transfection for 24 h, 48 h, and 72 h (**Figure 2A**). The results showed that the vivo-3′UTR of four concentrations significantly inhibited dengue virus RNA load at 24-h transfection, and the higher the concentration, the lower the viral RNA load of the dengue virus (**Figure 2B**). Compared with that in the NT group, the relative viral RNA load in the 0.01 μM group decreased by 84.46%, the 0.1 μM group decreased by 97.48%, the 1 μM group decreased by 99.58%, and the 10 μM group decreased by 99.97% (**Figure 2C**). In addition, the results of viral inhibition at different infection times showed that the inhibition rate of viral RNA in the 1 μM group was the highest at 48 h of virus infection, and the inhibition rate of viral RNA in the 10 μM group was the highest at 72 h of virus infection (**Figure 2D**). At the same time, to exclude the influence of different concentrations of vivo-MOs on cell growth vitality, we conducted a CCK-8 cell viability experiment, and the results showed that the cell viability slightly increased with the vivo-MO added (**Supplementary Figure S2**). However, there was no statistical difference in cell activity between the experimental groups and the control group at the same time point, indicating that different concentrations of drugs had no significant effect on cell viability.

### Screening of optimal administration time and concentration of the vivo-3′UTR

To evaluate the effects of different vivo-3′UTR administration times on viral inhibition, three experimental groups were designed with different administration times and the same drug treatment concentration. A 10-μM concentration of vivo-3′UTR was added at 2 h, 6 h, and 12 h after virus infection. According to the time of virus infection, the expression of dengue virus RNA in cells was measured in 24 h, 48 h, and 72 h. The results showed that the earlier the time of administration after virus infection, the higher the inhibition rate of the drug against the dengue virus (**Figure 2E**). After 72 h of viral infection, compared with that in the NT group, the relative viral RNA load in the 2-h group was reduced by 99.94%, the relative viral RNA load in the 6-h group was reduced by 99.82%, and the relative viral RNA load in the 12-h group was reduced by 98.78% (**Figure 2F**). The inhibition curve, based on varying drug administration times, indicated that the highest inhibition rate of the dengue virus was observed at 48 h post-infection in the group that received the drug at 2 h post-infection (**Figure 2G**). These results indicate that the optimal inhibitory effect on the virus was observed when vivo-MO was administered 2 h post-infection. This timing likely corresponds to the early stage of dengue virus replication, during which the addition of the drug may significantly impact the processes of mRNA transcription and translation.

**Figure 3:**
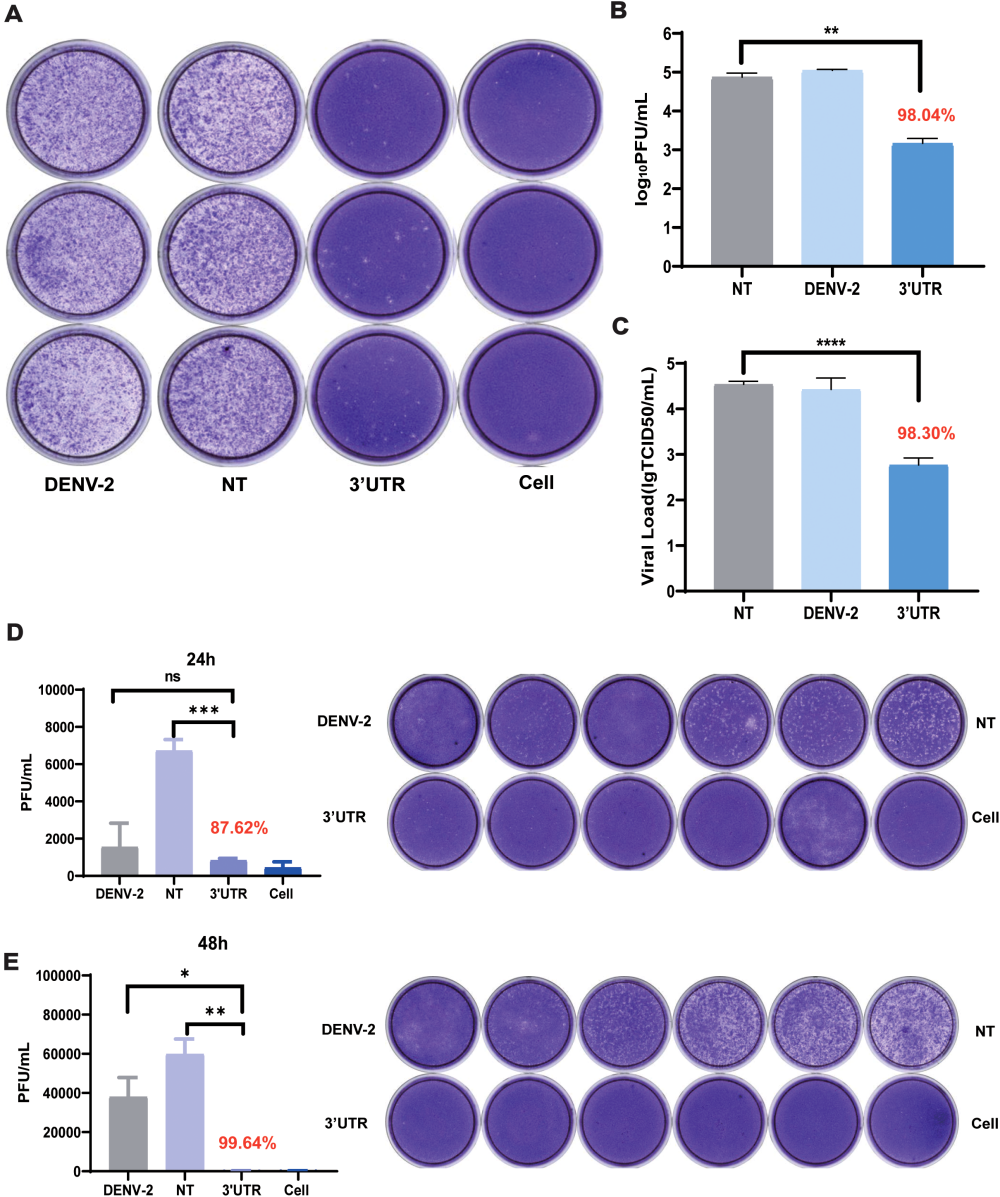
Plaque assay and TCID50 assay to verify the vivo-3′UTR anti-dengue effect. **(A, B)** Plaque assay to verify vivo-3′UTR anti-dengue effect in DENV-2 infection at 72 h. **(C)** TCID50 to verify vivo-3′UTR anti-dengue effect in DENV-2 infection at 72 h. **(D, E)** Plaque assay to verify vivo-3′UTR anti-dengue effect in DENV-2 infection at 24 h **(D)** and 48 h **(E)**. n = 3 for each group, two-tailed Student’s t-test; *p < 0.05; **p < 0.01; ***p < 0.001; ****p < 0.0001; ns, no significant difference; bars represent mean ± SD. DENV, dengue virus.

To evaluate the inhibitory effect of vivo-3′UTR on the infection of live virus, the effect of this target on the live viral titers of dengue virus was examined using a plaque assay and TCID50. The experimental results showed that vivo-3′UTR could significantly inhibit viral infection. Compared with that of the NT group, the dengue virus titer was reduced by 98.04% after 72 h of infection, and the vivo-3′UTR effects were greater than a 2-log10 reduction in DENV-2 titers at day 3 (**Figures 3A, B**). TCID50 was used to verify the vivo-3′UTR anti-dengue effect in DENV viral infection at 72 h; the dengue virus titer was reduced by 98.30% after 72 h of infection (**Figure 3C**). Meanwhile, the plaque assay experimental results showed that, compared with those in the NT group, the vivo-3′UTR effects kept the DENV-2 titer consistently below the cutoff value (1,000 PFU/mL) on days 1 and 2 (**Figures 3D, E**).

### The effect of vivo-3′UTR pre-treatment and post-treatment for inhibition rate

**Figure 4:**
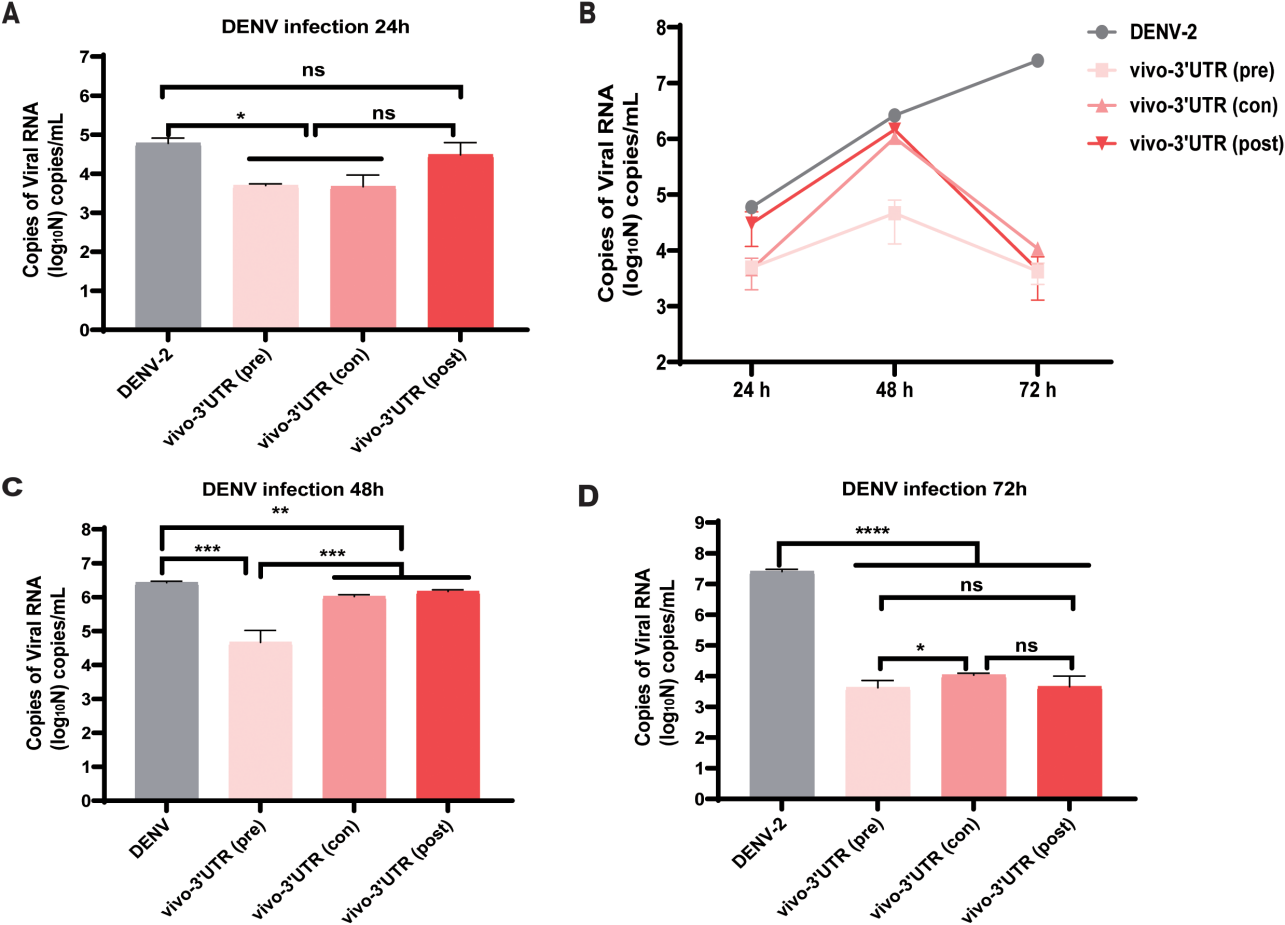
The antiviral efficacy evaluation of vivo-3′UTR under three conditions: pre-treatment, post-treatment, and con-treatment. **(A–D)** Pre-treatment, post-treatment, and con-treatment anti-dengue effect of vivo-3′UTR in DENV-2 infection at 24 h **(A)**, 48 h **(C)**, and 72 h **(D)**. Pre (pre-treatment) refers to the addition of vivo-3′UTR at 24 h before dengue virus infection. Con (concurrent treatment) refers to the concurrent addition of vivo-3′UTR when dengue virus infection. Post (post-treatment) refers to the addition of vivo-3′UTR 2 h after dengue virus infection. n = 3 for each group, two-tailed Student’s t-test; *p < 0.05; **p < 0.01; ***p < 0.001; ****p < 0.0001; ns, no significant difference; bars represent mean ± SD. DENV, dengue virus.

Then, we explored the treatment effects of added vivo-3′UTR before and after dengue infection and the treatment effects of added vivo-3′UTR at infection of the dengue virus concurrently. The results showed that, compared with that of the untreated (DENV) group, the inhibition rate of dengue viral RNA load was significantly higher than that of the other two groups when vivo-3′UTR was added 24 h earlier, indicating that vivo-3′UTR added in advance played a preventive role in the treatment of viral infection (**Figures 4A, B**). The results at 48 h showed that with the extension of vivo-3′UTR addition time, the vivo-3′UTR pre group constituted greater than a 2-log10 reduction in DENV-2 titers at 48 h (**Figure 4C**). In addition, the results at 72 h showed that with the extension of vivo-3′UTR addition time, the viral RNA load of dengue virus in the three experimental groups could be significantly reduced, and the inhibition rates were 99.98%, 99.96%, and 99.98%; the vivo-3′UTR effects constituted a 4-log10 average reduction in DENV-2 titers at day 3 (**Figure 4D**). These results indicated that vivo-MO targeting dengue 3′UTR can play a good preventive role before virus infection and still has a good therapeutic effect during and after virus infection (**Figure 4B**).

### The inhibition rate of vivo-3′UTR for different DENV serotypes

**Figure 5:**
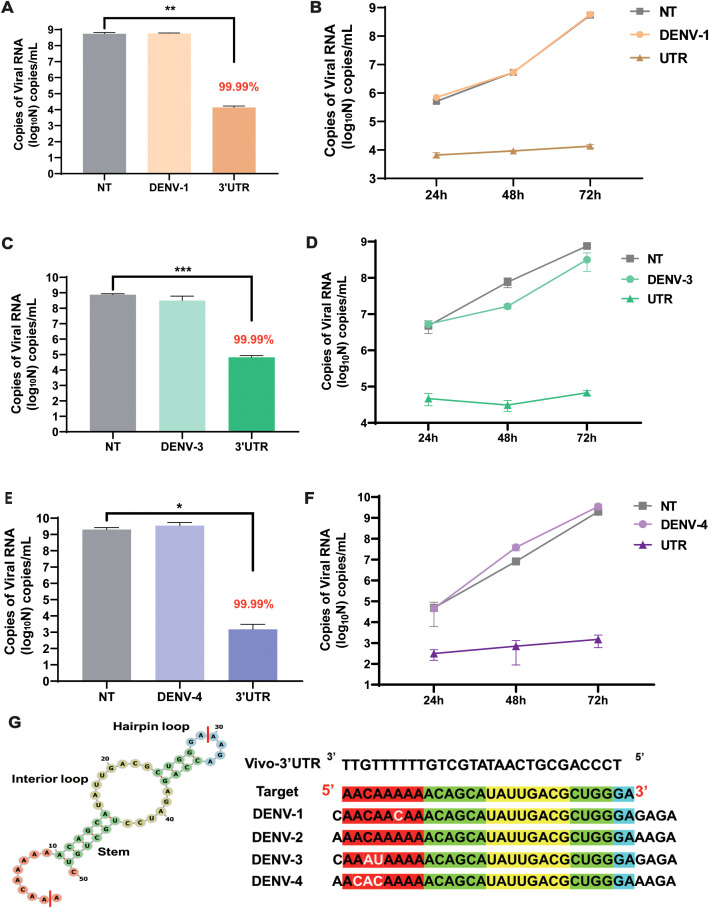
The inhibitory effect of vivo-3′UTR on different serotypes of dengue virus (DENV-1, DENV-3, and DENV-4). **(A, B)** The vivo-3′UTR can effectively inhibit the replication of DENV-1 after viral infection at 24 h, 48 h, and 72 h **(A)**. **(C, D)** The vivo-3′UTR can effectively inhibit the replication of DENV-3 after viral infection at 24 h, 48 h, and 72 h **(D)**. **(E, F)** The vivo-3′UTR can effectively inhibit the replication of DENV-4 after viral infection at 24 h, 48 h, and 72 h **(F)**. n = 3 for each group, two-tailed Student’s t-test; *p < 0.05; **p < 0.01; ***p < 0.001, bars represent mean ± SD. **(G)** The genome sequences of some dengue virus serotypes were aligned with the targeting sequence of vivo-3′UTR. DENV, dengue virus.

To evaluate the inhibitory effect of this target on different serotypes of the dengue virus in cells, vivo-3′UTR was used to inhibit the dengue virus in other three serotypes (DENV-1, DENV-3, and DENV-4). The results showed that the viral RNA load of the NT group was 5 × 10^8^ copies/mL after DENV-1 infection at 72 h, and the viral RNA load of the vivo-3′UTR group was only 10^4^ copies/mL; the results indicated that the viral RNA load of DENV-1 in the vivo-3′UTR group was reduced by 99.99% compared with that in the NT group (**Figure 5A**). The vivo-3′UTR effects constituted greater than a 4-log10 reduction in DENV-1 viral RNA load at day 3 (**Figure 5B**). In the antiviral experiment against DENV-3, the viral RNA load of both the control group and the NT group was 6 × 10^8^ copies/mL, and the viral RNA load of the vivo-3′UTR group was 10^5^ copies/mL; the results indicated that vivo-3′UTR reduced the viral RNA load of DENV-3 by 99.99% compared with that in the NT group (**Figure 5C**). The vivo-3′UTR effects constituted greater than a 3-log10 reduction in DENV-3 viral RNA load at day 3 (**Figure 5D**). In the antiviral experiment against DENV-4, the viral RNA load of both the control group and the NT group reached 2 × 10^9^ copies/mL, and the viral RNA load of vivo-3′UTR was 1 × 10^3^ copies/mL after infection 72 h; the results indicated that vivo-3′UTR still reduced the viral RNA load of DENV-4 by 99.99% compared with that in the NT group (**Figure 5E**). The vivo-3′UTR effects constituted greater than a 6-log10 reduction in DENV-4 viral RNA load at day 3 (**Figure 5F**). This proves that the vivo-3′UTR screened in this study has a good inhibitory effect on all serotypes of the dengue virus.

Then, we predicted the secondary structure of 3′UTR sequences using RNAfold (**Figure 5G**; **Supplementary Figure S3**); Vivo-3′UTR targets the neck-loop regions (including the hairpin loop, interior loop, and stem) of the dengue virus untranslated region (**Supplementary Figure S3**). Concurrently, the target sequences were aligned with the reference sequences of the four dengue virus serotypes. The alignment results indicated a 1-base mismatch with the DENV-1 sequence, a 2-base mismatch with the DENV-3 sequence, and a 3-base mismatch with the DENV-4 sequence (**Figure 5G**; **Supplementary Figure S4**). None of these mismatches, situated within the single-stranded region of the untranslated region, impacted the inhibitory efficacy of vivo-3′UTR against various serotypes of dengue viruses. Furthermore, to assess the inhibitory efficacy of vivo-3′UTR against dengue virus across various cell lines, we conducted an evaluation specifically in HepG2 cells. The findings indicated that the viral RNA load in the NT group was 10^12^ copies/mL. The viral RNA load of the vivo-3′UTR group was 10^7^ copies/mL, the viral RNA load decreased by 10^5^ times, and the inhibition rate of dengue virus in the vivo-3′UTR group reached 99.99% after 72 h of viral infection (**Supplementary Figure S5**). It was proved that vivo-3′UTR could effectively inhibit dengue virus infection in different cells.

## Discussion

Dengue remains the most prevalent and rapidly spreading mosquito-borne viral disease affecting humans (29). In recent decades, the swift dissemination of the dengue virus across various nations has led to an increase in the incidence of dengue fever, marked by a high prevalence of diverse dengue virus serotypes in numerous tropical regions. Finding and discovering effective drug targets is the key to the current fight against the dengue virus. The early researchers (25) designed three PMOs targeting the DENV and analyzed its mechanism of action. The results reveal that 3′SL-targeted PPMO inhibited DENV replication by interfering with both mRNA transcription and protein translation machinery, which is consistent with our results (25). The 5′SL PMO inhibited viral replication by interfering with the translation process, and 3′CS blocked viral RNA synthesis, whereas 3′SL acted both ways (25). In this study, we obtained the most conserved sequences of DENV from 10 different gene regions and found that vivo-MO targeting the 3′UTR of the dengue virus has a higher effective inhibition effect on the dengue virus compared to vivo-MO targeting the other nine genes of the dengue virus. The targeting sequence obtained differs from the targeting position reported by Phumesin et al. (vivo-MO-1) (26). The vivo-MO-1 sequence is complementary with the 10,674 to 10,698 nucleotides base of the DENV-2 genome (NCBI GenBank, NC_001474.2) (26); our sequence of the vivo-3′UTR is complementary with the 10609 to 10636. However, this is consistent with the target gene (UTR region) of previous studies, which further indicates that the 3′UTR gene plays a key role in dengue virus replication. In further analysis, we guessed that targeting this region may inhibit the function of the 3′UAG termination codon of the translated polyprotein of the dengue virus, affecting the assembly of the dengue virus and thereby inhibiting the replication of the dengue virus.

Furthermore, we analyzed the genetic sequences of four serotypes of the dengue virus and found that the untranslated region is one of the most conserved gene regions of the dengue virus, and it has various functions in the initiation and regulation of viral translation, replication, and assembly. The vivo-3′UTR targeting the dengue virus can fully overlap with the 3′UTR region of dengue virus type 2, but there is a 2–3 base mismatch with the 3′UTR gene region of the dengue virus in the other three serotypes (**Supplementary Figure S4**). Nevertheless, in the experiment, the inhibition rate of vivo-3′UTR against the other three serotypes could still reach 99%, which further suggests that the vivo-3′UTR sequence designed by us has a broad inhibition effect on different strains of the dengue virus. In addition, we analyzed the antiviral efficacy of vivo-MO in both pre-treatment and post-treatment conditions, and we analyzed the influence of different times of administration after infection on the inhibition efficiency of the dengue virus, providing an idea of drug delivery and a potentially effective drug target for the subsequent realization of efficient inhibition of dengue virus in animal models.

The use of oligonucleotides is considered a very attractive means of inhibiting viral replication. Compared to DNA, mRNA is ubiquitous and more likely to be manipulated, which has led to antisense oligonucleotides that target mRNA or microRNA and have been widely used as a research tool in molecular biology to specifically and selectively downregulate gene expression (30). Traditional small molecule drugs mainly act on pathogenic proteins, while antisense oligonucleotide drugs directly act on pathogenic genes themselves, so they are more selective than small molecule chemical drugs and have the advantages of high efficiency, low toxicity, stability and effectiveness, and not easy to produce drug resistance (31). The structure of MO determines that it has high stability and can be used in *in vivo* and *in vitro* experiments without chemical modification. In addition, MO can add functional groups at both ends of the sequence to meet different research needs. The *in vivo* delivery moiety is an octa-guanidine dendrimer, which conjugates to the 3′ end of a morpholino oligo to enhance cell penetration, and the 5′ end of Vivo-Morpholinos can be further modified to achieve more features. Another major advantage of oligonucleotides is that their design is relatively simple and rational, and they only bind to specific nucleic acid sequences or protein targets. At the same time, through simple modification of the sequence, different genes can be targeted, and it has good programmability. Of course, the low molecular weight of oligonucleotides also makes them a valuable alternative to antiviral drugs. In this study, 3′UTR may be used as a candidate target region for subsequent development of other nucleotide anti-dengue virus drugs, and vivo-3′UTR of targeting 3′UTR may be further developed as a mature anti-dengue virus ASO drug. However, this study did not assess the antiviral effect, biological toxicity, or *in vivo* half-life of vivo-3′UTR in mouse models. Additionally, vivo-MO, which does not require a transport carrier, exposes its oligonucleotide sequence within cells, leading to a short retention time in the body. In the future, it may undergo further modifications to mitigate the degradation effects of intracellular nucleases and extend its half-life.

## Data Availability

The raw data supporting the conclusions of this article will be made available by the authors, without undue reservation.
