# Supplementary material for: Effective inhibition of dengue virus replication using 3′UTR-targeted Vivo-Morpholinos
Source: Front Immunol. 2024 Nov 29;15:1491230. doi: 10.3389/fimmu.2024.1491230 (PMC11638040; doi:10.3389/fimmu.2024.1491230)
Supplement: Supplementary file 1 [file DataSheet1.docx]

***Supplementary Material***

1. **Supplementary Figures and Tables**
   1. **Supplementary Figures**

**Figure S1.** Structure schematic of the 10 vivo-MO.

(A) Antisense oligonucleotide terminal modification structure. (B) The modification and connection of the antisense oligonucleotide group.

**
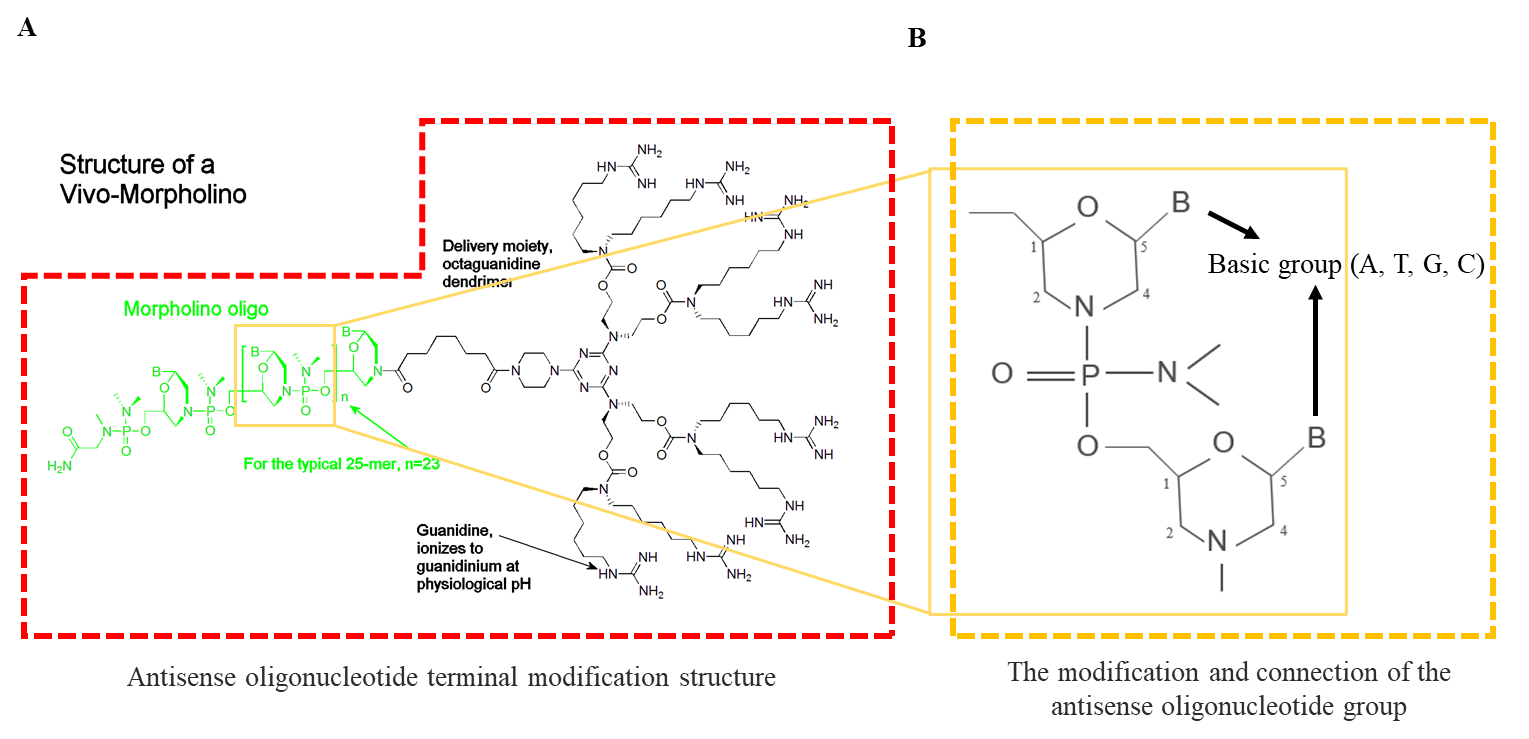
**

**Figure S2.** Toxicity test of cell.

(A) Toxicity test of vivo-MO of four concentrations gradient (0.01, 0.1, 1, 10 μM) on Vero cell. Cell viability was measured by Cell counting Kit-8 (Beyotime).

**Figure S3.** Prediction of the secondary structure of 3’UTR sequences using RNAfold.


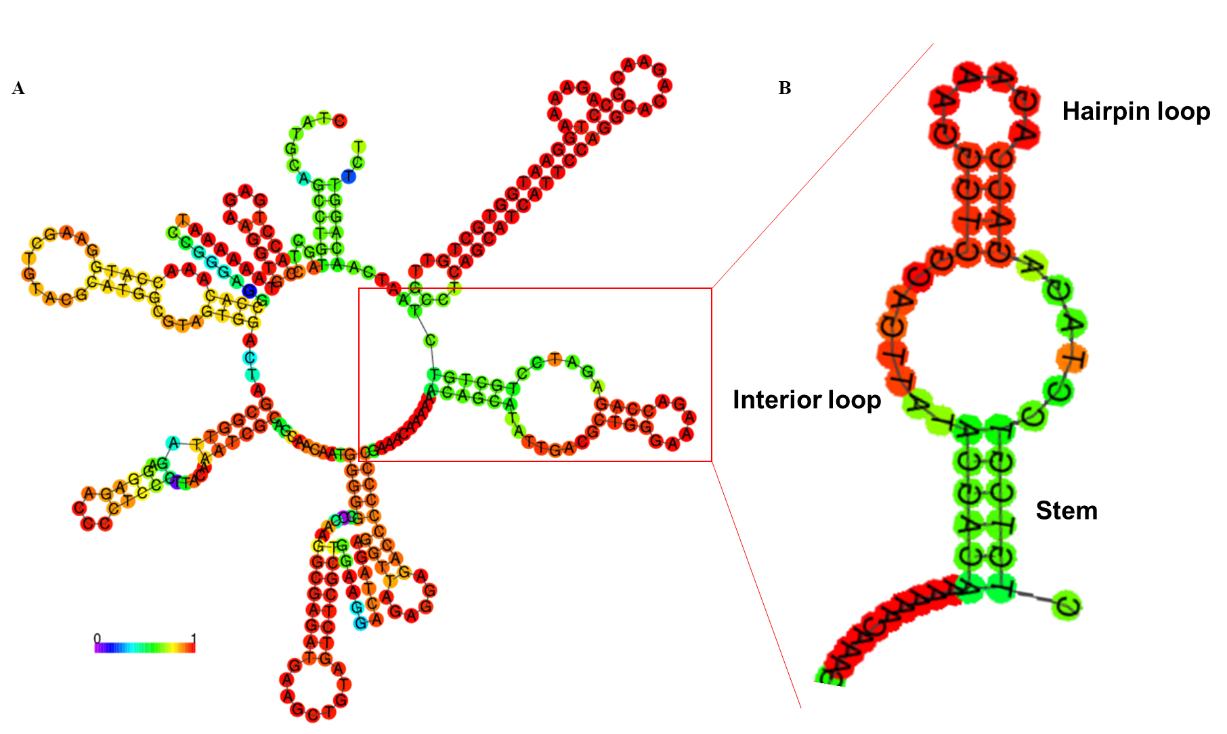
(A) Secondary structure of the DENV-2 3’UTR sequence. (B) Secondary structure of the vivo-3’UTR target sequence.

**Figure S4.** Results of sequence comparison between different serotypes of Dengue virus and antiviral targets.


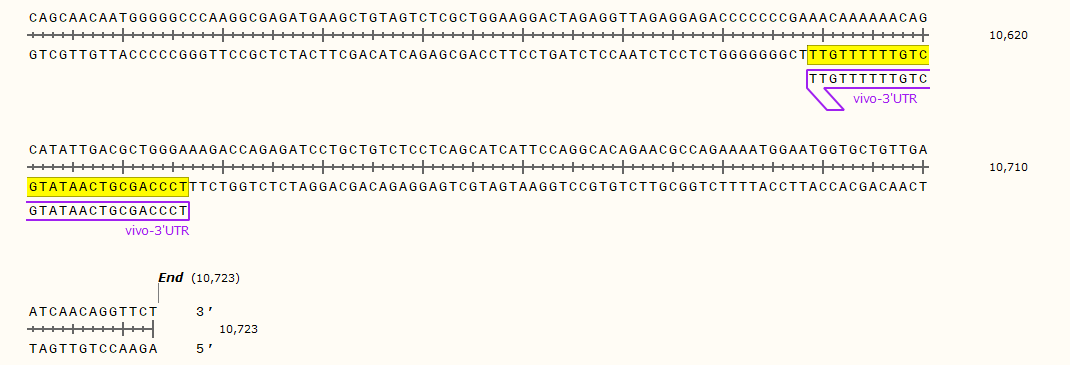
dengue virus type 2 (NCBI GenBank, NC_001474.2)


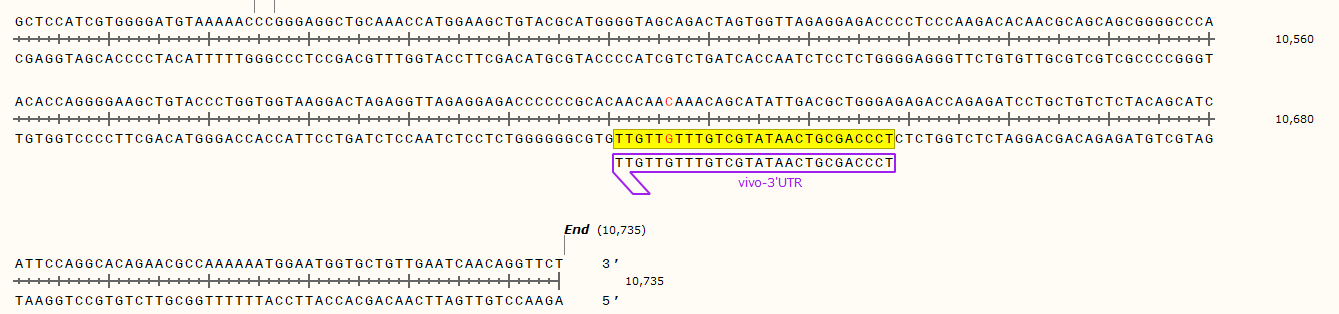
dengue virus type 1 (NCBI GenBank, NC_001477.1)


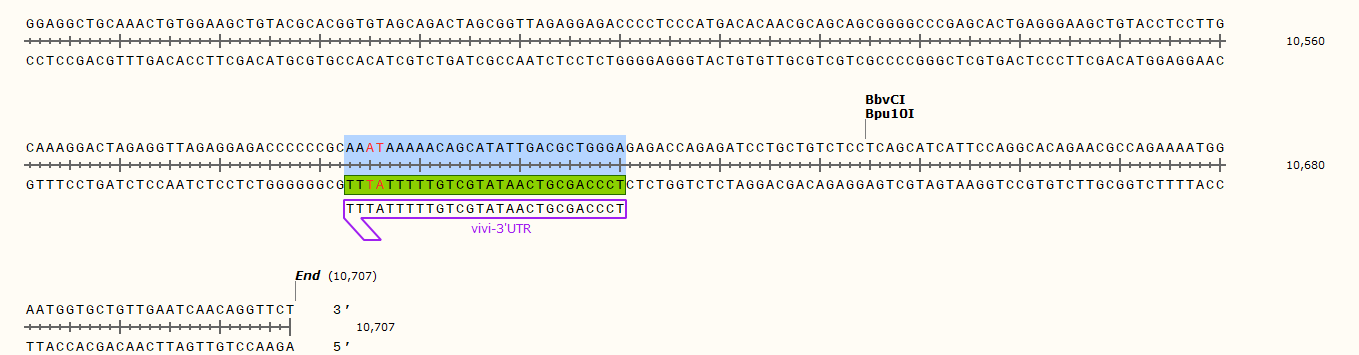
dengue virus type 3 (NCBI GenBank, NC_001475.2)


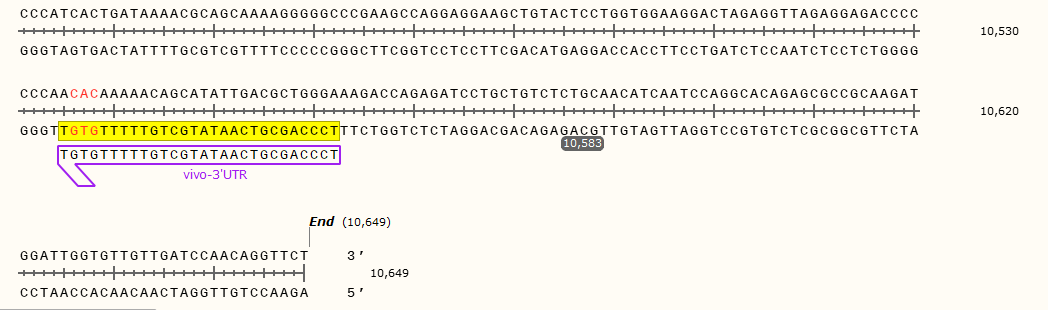
dengue virus type 4 (NCBI GenBank, NC_002640.1)

**Figure S5.** The inhibitory efficiency of vivo-3 'UTR in different cell lines.

**
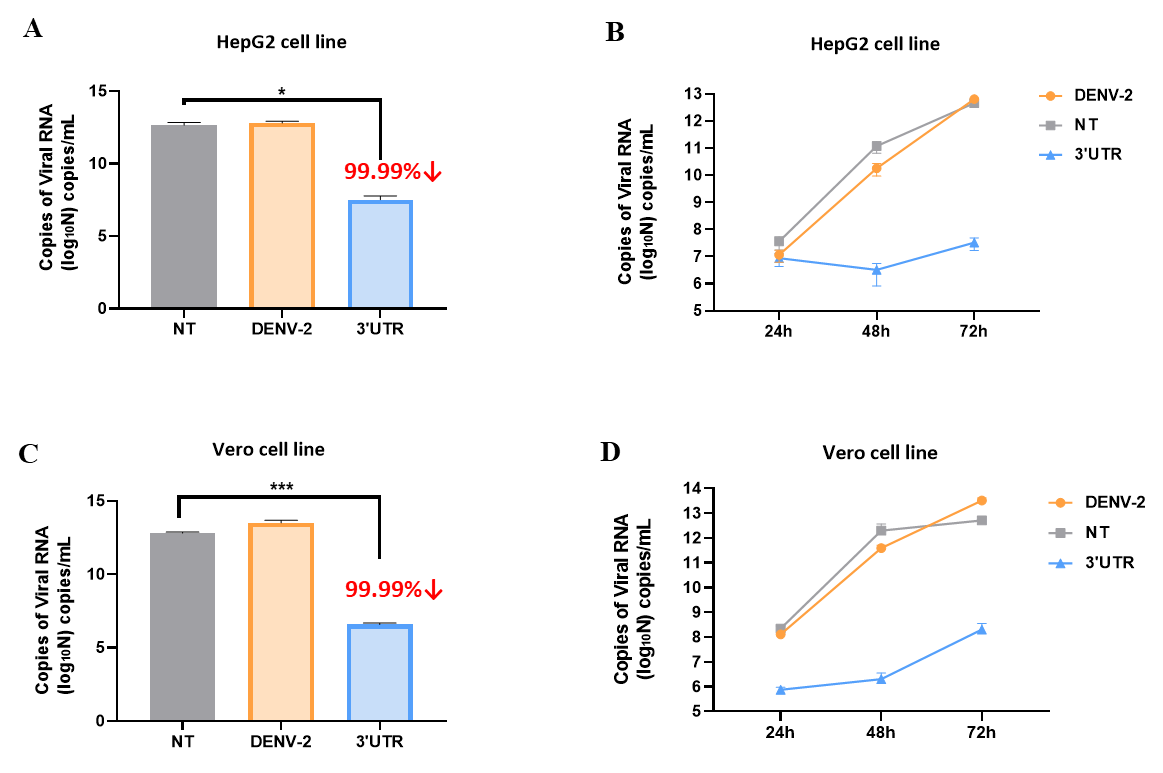
**(A and B) Anti-dengue effect of vivo-3 'UTR in HepG2 cells. (C and D) Anti-dengue effect of vivo-3 'UTR in Vero cells. (B and D) Anti-dengue effect of vivo-3 'UTR in DENV viral infection 24h, 48h and 72h. (n=3 technical replicates; *, p < 0.05; ***, p < 0.001; ns, no significant difference; bars represent mean ± s.e.m.).

- 1. **Supplementary Tables**

**Supplementary Table 1** The oligonucleotides sequences used in this study.

| **Target name** | **Oligonucleotides sequence (5’-3’)** | **Targeted protein type** | **Targeted DENV** **sequence site** |
| --- | --- | --- | --- |
| vivo-C | CGGTTTCTCTCGCGTTTCAGCATATTGA | Structural protein Capsid | 133-160nt |
| vivo-PrM | CCAGGCCCCTTCTGATGACATCCATGTT | Structural protein pre membrane | 762-789nt |
| vivo-NS1 | TTGTATTGTTCTGTCCATGTGTGCACGT | Nonstructural protein 1 | 2492-2519nt |
| vivo-NS2a | TGCAACTCACTTTCCATGCGTTTTGTAA | Nonstructural protein 2a | 3958-3985nt |
| vivo-NS2b | ACTAATGGTCCTGTCATGGGAATATCAT | Nonstructural protein 2b | 4202-4229nt |
| vivo-NS3 | CCACGTGTGACATGCCACATTGTATGGA | Nonstructural protein 3 | 4658-4685nt |
| vivo-NS4a | GTCAGAGACTTTCTTCCGGCTGCAAATT | Nonstructural protein 4a | 6356-6383nt |
| vivo-NS4b | CTTTTCTGAGCTTCTCTGGTTGCTTTTG | Nonstructural protein 4b | 7205-7232nt |
| vivo-NS5 | TCTCTTTTTCCCATCATGTTGTACACAC | Nonstructural protein 5 | 8918-8945nt |
| vivo-3’UTR | TCCCAGCGTCAATATGCTGTTTTTTGTT | 3’ Untranslated Region | 10609-10636nt |
| vivo-NT | CCTCTTACCTCAGTTACAATTTATA | Non-targeted contrast |  |
